# Supplementary material for: A fault-intrusion-tolerant system and deadline-aware algorithm for scheduling scientific workflow in the cloud
Source: PeerJ Comput Sci. 2021 Nov 2;7:e747. doi: 10.7717/peerj-cs.747 (PMC8576554; doi:10.7717/peerj-cs.747)
Supplement: Supplemental Information 6 [file peerj-cs-07-747-s006.zip › Myproject1.1/docs/org/cloudbus/cloudsim/power/class-use/PowerVmSelectionPolicy.html]

Uses of Class org.cloudbus.cloudsim.power.PowerVmSelectionPolicy


JavaScript is disabled on your browser.


- Overview
- Package
- Class
- Use
- Tree
- Deprecated
- Index
- Help

- Prev
- Next

- Frames
- No Frames

- All Classes

## Uses of Class org.cloudbus.cloudsim.power.PowerVmSelectionPolicy

- Packages that use PowerVmSelectionPolicy

  | Package | Description |
  | --- | --- |
  |  |  |
  | --- | --- |
  | org.cloudbus.cloudsim.examples.power |  |
  | org.cloudbus.cloudsim.power |  |
- - ### Uses of PowerVmSelectionPolicy in org.cloudbus.cloudsim.examples.power

    Methods in org.cloudbus.cloudsim.examples.power that return PowerVmSelectionPolicy

    | Modifier and Type | Method and Description |
    | --- | --- |
    |  |  |
    | --- | --- |
    | `protected PowerVmSelectionPolicy` | RunnerAbstract.`getVmSelectionPolicy(java.lang.String vmSelectionPolicyName)` Gets the vm selection policy. |
  - ### Uses of PowerVmSelectionPolicy in org.cloudbus.cloudsim.power

    Subclasses of PowerVmSelectionPolicy in org.cloudbus.cloudsim.power

    | Modifier and Type | Class and Description |
    | --- | --- |
    |  |  |
    | --- | --- |
    | `class` | `PowerVmSelectionPolicyMaximumCorrelation` The Maximum Correlation (MC) VM selection policy. |
    | `class` | `PowerVmSelectionPolicyMinimumMigrationTime` The Minimum Migration Time (MMT) VM selection policy. |
    | `class` | `PowerVmSelectionPolicyMinimumUtilization` The Minimum Utilization (MU) VM selection policy. |
    | `class` | `PowerVmSelectionPolicyRandomSelection` The Random Selection (RS) VM selection policy. |

    Methods in org.cloudbus.cloudsim.power that return PowerVmSelectionPolicy

    | Modifier and Type | Method and Description |
    | --- | --- |
    |  |  |
    | --- | --- |
    | `PowerVmSelectionPolicy` | PowerVmSelectionPolicyMaximumCorrelation.`getFallbackPolicy()` Gets the fallback policy. |
    | `protected PowerVmSelectionPolicy` | PowerVmAllocationPolicyMigrationAbstract.`getVmSelectionPolicy()` Gets the vm selection policy. |

    Methods in org.cloudbus.cloudsim.power with parameters of type PowerVmSelectionPolicy

    | Modifier and Type | Method and Description |
    | --- | --- |
    |  |  |
    | --- | --- |
    | `void` | PowerVmSelectionPolicyMaximumCorrelation.`setFallbackPolicy(PowerVmSelectionPolicy fallbackPolicy)` Sets the fallback policy. |
    | `protected void` | PowerVmAllocationPolicyMigrationAbstract.`setVmSelectionPolicy(PowerVmSelectionPolicy vmSelectionPolicy)` Sets the vm selection policy. |

    Constructors in org.cloudbus.cloudsim.power with parameters of type PowerVmSelectionPolicy

    Constructor and Description

    `PowerVmAllocationPolicyMigrationAbstract(java.util.List<? extends Host> hostList,
    PowerVmSelectionPolicy vmSelectionPolicy)`

    Instantiates a new power vm allocation policy migration abstract.

    `PowerVmAllocationPolicyMigrationInterQuartileRange(java.util.List<? extends Host> hostList,
    PowerVmSelectionPolicy vmSelectionPolicy,
    double safetyParameter,
    PowerVmAllocationPolicyMigrationAbstract fallbackVmAllocationPolicy)`

    Instantiates a new power vm allocation policy migration mad.

    `PowerVmAllocationPolicyMigrationInterQuartileRange(java.util.List<? extends Host> hostList,
    PowerVmSelectionPolicy vmSelectionPolicy,
    double safetyParameter,
    PowerVmAllocationPolicyMigrationAbstract fallbackVmAllocationPolicy,
    double utilizationThreshold)`

    Instantiates a new power vm allocation policy migration mad.

    `PowerVmAllocationPolicyMigrationLocalRegression(java.util.List<? extends Host> hostList,
    PowerVmSelectionPolicy vmSelectionPolicy,
    double safetyParameter,
    double schedulingInterval,
    PowerVmAllocationPolicyMigrationAbstract fallbackVmAllocationPolicy)`

    Instantiates a new power vm allocation policy migration local regression.

    `PowerVmAllocationPolicyMigrationLocalRegression(java.util.List<? extends Host> hostList,
    PowerVmSelectionPolicy vmSelectionPolicy,
    double safetyParameter,
    double schedulingInterval,
    PowerVmAllocationPolicyMigrationAbstract fallbackVmAllocationPolicy,
    double utilizationThreshold)`

    Instantiates a new power vm allocation policy migration local regression.

    `PowerVmAllocationPolicyMigrationLocalRegressionRobust(java.util.List<? extends Host> hostList,
    PowerVmSelectionPolicy vmSelectionPolicy,
    double safetyParameter,
    double schedulingInterval,
    PowerVmAllocationPolicyMigrationAbstract fallbackVmAllocationPolicy)`

    Instantiates a new power vm allocation policy migration local regression.

    `PowerVmAllocationPolicyMigrationLocalRegressionRobust(java.util.List<? extends Host> hostList,
    PowerVmSelectionPolicy vmSelectionPolicy,
    double safetyParameter,
    double schedulingInterval,
    PowerVmAllocationPolicyMigrationAbstract fallbackVmAllocationPolicy,
    double utilizationThreshold)`

    Instantiates a new power vm allocation policy migration local regression.

    `PowerVmAllocationPolicyMigrationMedianAbsoluteDeviation(java.util.List<? extends Host> hostList,
    PowerVmSelectionPolicy vmSelectionPolicy,
    double safetyParameter,
    PowerVmAllocationPolicyMigrationAbstract fallbackVmAllocationPolicy)`

    Instantiates a new power vm allocation policy migration mad.

    `PowerVmAllocationPolicyMigrationMedianAbsoluteDeviation(java.util.List<? extends Host> hostList,
    PowerVmSelectionPolicy vmSelectionPolicy,
    double safetyParameter,
    PowerVmAllocationPolicyMigrationAbstract fallbackVmAllocationPolicy,
    double utilizationThreshold)`

    Instantiates a new power vm allocation policy migration mad.

    `PowerVmAllocationPolicyMigrationStaticThreshold(java.util.List<? extends Host> hostList,
    PowerVmSelectionPolicy vmSelectionPolicy,
    double utilizationThreshold)`

    Instantiates a new power vm allocation policy migration mad.

    `PowerVmSelectionPolicyMaximumCorrelation(PowerVmSelectionPolicy fallbackPolicy)`

    Instantiates a new power vm selection policy maximum correlation.

- Overview
- Package
- Class
- Use
- Tree
- Deprecated
- Index
- Help

- Prev
- Next

- Frames
- No Frames

- All Classes
